# Leptospirosis meningitis transmission from a pet mouse: a case report

**DOI:** 10.1186/s13256-019-2265-7

**Published:** 2019-11-28

**Authors:** Anne Christine Nordholm, Lars Haukali Omland, Steen Villumsen, Imad Al-Subeihe, Terese L. Katzenstein

**Affiliations:** 1 Department of Infectious Diseases, Copenhagen University Hospital Rigshospitalet, Blegdamsvej 9, 2100 Copenhagen, Denmark; 2 Department of Internal Medicine Q, Frederiksberg Hospital, Frederiksberg, Denmark; 3 Department of Clinical Medicine, University of Copenhagen, Copenhagen, Denmark

**Keywords:** Leptospirosis, Meningitis, Zoonosis, Emerging disease, Case report

## Abstract

**Background:**

Leptospirosis is a reemerging zoonosis with a worldwide distribution and a wide range of clinical manifestations. We report a case of leptospirosis meningitis in a previously healthy woman infected by her pet mouse.

**Case presentation:**

A 27-year-old Caucasian woman with pet mice presented to our institute with a 1 week history of fever, headache, myalgia, vomiting, diarrhea, and dark urine. Her admission examination revealed neck stiffness, conjunctivitis, and icteric sclera. Her liver enzymes, bilirubin, white blood cell count, and C-reactive protein were elevated. Her cerebrospinal fluid showed an elevated white blood cell count. Polymerase chain reactions using her cerebrospinal fluid, blood, and urine showed negative results for leptospirosis, but the result of her microagglutination test was positive for *Leptospira interrogans* serovar *sejroe* with a more than threefold increase in paired sera. The patient was treated with ceftriaxone for 1 week, and her condition steadily improved.

**Conclusions:**

This case report raises awareness of pet rodents as sources of leptospirosis. Leptospirosis meningitis should be considered in patients with meningeal symptoms and pet rodents.

## Background

Leptospirosis is a reemerging zoonosis that occurs worldwide and is believed to be underdiagnosed because of challenging diagnostics and a wide clinical spectrum of disease [1]. The incidence in Europe has recently been reported to be 0.13 per 100,000 inhabitants [2]. Leptospirosis is caused by *Leptospira* species, a group of spirochete bacteria [3] with 29 described serogroups and more than 200 different *Leptospira* serovars [4]. *Leptospira* species infect mammals as well as fish, birds, and reptiles [5]. Infected animals become reservoirs for the disease, and rodents constitute the most important reservoir [6]. *Leptospira* species colonize the renal proximal tubules of their reservoir hosts and are excreted in the urine [7, 8]. When *Leptospira* are excreted into the environment, they are able to survive for several months in water [9], which constitutes an important source of infection [4]. Humans are usually infected by contact with urine-contaminated water [10]. *Leptospira* enter the human body by penetration of damaged skin or via oral, genital, or conjunctival mucous membranes, and they are hematogenously disseminated afterward [11]. Disease manifestations vary from mild or asymptomatic to severe illness with multiorgan failure [12]. Patients with leptospirosis typically present with fever, headache, and myalgia [11], but symptoms of any organ may be apparent [10]. Severe forms include meningitis, pulmonary hemorrhage with respiratory failure, or Weil’s disease characterized by jaundice, bleeding, and renal failure [11]. Leptospirosis may mimic other infectious diseases, such as influenza, viral hepatitis, brucellosis, infectious mononucleosis, malaria, or dengue, depending on the setting [10], or even bacterial or viral meningitis [13]. A recent review on leptospirosis meningitis revealed that almost all patients (N = 366 adults) presented with fever (98%), headache (94%), and neck stiffness (93%) [13]. The authors of that review found that the patients’ median age was 33 years, that most were male, and that the majority were believed to have acquired the infection from their work environment, with others contracting it after contact with fresh water [13]. There were no cases of transmission from pets. In this report, we describe a case of leptospirosis meningitis in a young, previously healthy woman who was most likely infected by her pet mouse.

## Case presentation

A 27-year-old Caucasian woman with an unremarkable medical history was admitted to a local hospital because of 1 week of fever, lower back pain, and cough. Since her fifth day of disease, she had experienced severe headache, ear and eye pain, and yellowish eyes. Further complaints included nausea, vomiting, dark urine, and diarrhea with pale stools. The patient had no recent travel history but had mice as pets, and one had fallen ill with conjunctivitis 1.5 months prior to the onset of the patient’s symptoms. Because of the patient’s symptoms and relevant exposure, leptospirosis and meningitis were suspected, and she was transferred to the Department of Infectious Diseases, Copenhagen University Hospital Rigshospitalet.

**Fig. 1:**
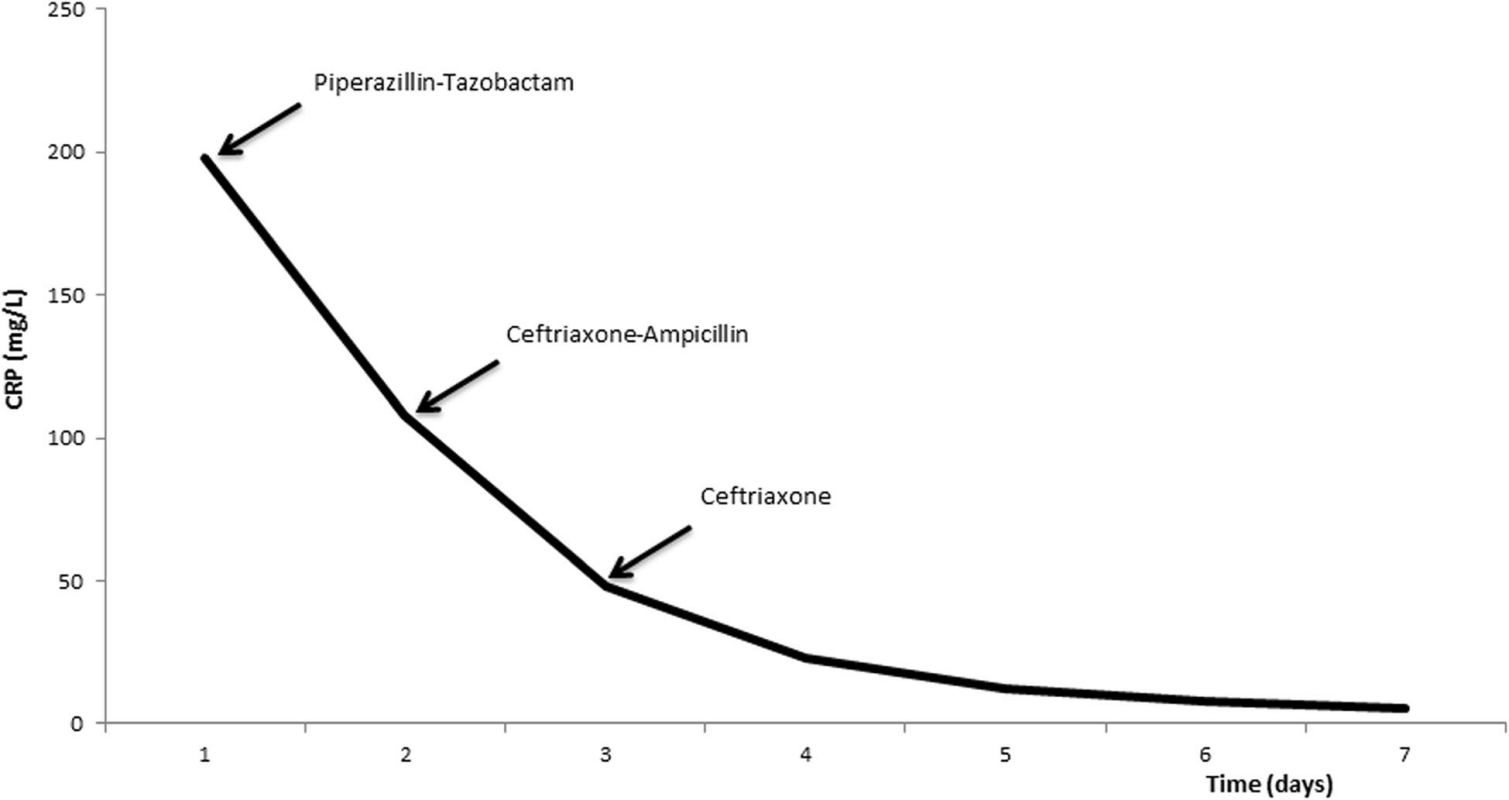
Antibiotic treatment and C-reactive protein during hospitalization

On admission, the patient’s physical examination revealed neck stiffness, conjunctivitis, and icteric sclera. Her blood pressure was 113/65 mmHg, heart rate was 79 beats/minute, temperature was 38.1 °C, respiratory rate was 16 breaths/minute, and oxygen saturation was 98% without oxygen supplementation. Her laboratory examination showed an elevated white blood cell count (WBC) of 12.3 × 10^9^/L and C-reactive protein (CRP) of 198 mg/L, along with increased liver function test values (alanine aminotransferase 186 U/L, alkaline phosphatase 359 U/L, γ-glutamyl transferase 624 U/L, and bilirubin 50 U/L) and hypoalbuminemia of 27 g/L. The results of the patient’s abdominal ultrasound and chest radiography were both normal. Cerebrospinal fluid (CSF) showed elevated leukocytes at 213 cells/mm^3^ (56% polymorphonuclear cells), lactic acid at 2.7 mmol/L, glucose at 3.4 mmol/L, and protein at 0.55 g/L. The result of her CSF culture was negative. Her blood and urine were examined on day 7 of disease with polymerase chain reaction (PCR) tests that showed negative results for leptospirosis. However, the result of her microagglutination test (MAT) on day 11 of disease was positive and demonstrated strongest reactivity against *Leptospira interrogans* serovar *sejroe* with antibody titers of 3000, increasing to 10,000 on day 22 of disease. Conventional blood cultures were collected before the antibiotic treatment was initiated, but urine culture and lumbar puncture were done after 1 day of treatment. The patient was initially empirically treated with piperacillin-tazobactam and then briefly shifted to a bacterial meningitis regimen with ampicillin and ceftriaxone. Because of suspected leptospirosis, the patient was treated with 2 g of ceftriaxone intravenously for 7 days, and her condition improved. After 1 week of hospitalization, the patient was discharged without any sequelae. Figure 1 illustrates the antibiotic treatment in relation to CRP level during 1 week of hospitalization.

## Discussion

We report a case of leptospirosis meningitis in a previously healthy young woman who was most likely infected by her pet mouse. In a recently published surveillance and outbreak report, six cases of leptospirosis transmission from pet rodents to humans were retrospectively recognized [14]. The median age of the patients was 31 years, and four of six cases were in women. One case was meningitis/meningoencephalitis [14]. A literature review revealed only four previous case reports about transmission of leptospirosis from pets [15–18], all of which were cases involving pet rats, and no cases were of meningitis. Though rare, these cases highlight the need for awareness of possible leptospirosis transmission from pets.

Leptospirosis has a wide range of disease manifestations and may mimic many other infectious diseases [10], which could potentially lead to misdiagnoses or doctor’s delay. Our patient had disseminated disease with symptoms arising from several organs. Leptospirosis was suspected because the patient had pet mice, and she was treated according to this suspicion. Prior to the onset of our patient’s symptoms, one of her pet mice had fallen ill with conjunctivitis. Unfortunately, the mouse was killed before being tested for leptospirosis. The incubation time for leptospirosis is 2–30 days [19], and the patients typically present with fevers and headache [11], which are also symptoms that could represent a central nervous system infection. Despite these frequent symptoms, meningitis is present in only around 20% of leptospirosis cases [13, 20]. According to the literature, leptospirosis meningitis often affects young adults with no serious concurrent medical diseases, and the prognosis is usually good [13]. In our patient’s case, CSF showed elevated WBC with a small predominance of polymorphonuclear cells, but we were not able to detect *Leptospira* in CSF by culture or real-time PCR, consistent with the literature [13, 21], nor did we succeed in detecting leptospires in blood or urine. The timing of the PCR test is very important for the identification of leptospires because they are detectable only in the blood and CSF during the first week and in the urine after 2–3 weeks of disease [10]. We might have conducted testing too late to detect *Leptospira* in the patient’s blood and CSF and too early to detect it in the urine. The advantage of PCR, which is widely used, is that it has both high sensitivity (100%) and specificity (93%) [12], though this varies, depending on the specific PCR assay used, the time when it is used in the disease course, and if it is used before antibiotics have been initiated. The downside of the PCR test is the narrow window of detectability in the disease course and the fact that the test does not provide information on *Leptospira* serogroup*.* The diagnostic “gold standard” is MAT [22], which measures antibody titers to specific *Leptospira* serovars [11]. Antibodies are usually not detectable until days 7–10 of disease [10]. An MAT result is considered positive when titers are > 100, and, more importantly, the diagnosis is confirmed if there is an approximately fourfold rise in titers in paired sera [11]. In our patient’s case, the clinical diagnosis was substantiated by the positive MAT result. Challenges regarding current diagnostics probably contribute to the disease’s being underreported, as stated by the World Health Organization [1]. In addition, serologic studies show much higher seropositive case rates than reported cases [20]. Only a fraction of patients with leptospirosis are hospitalized, because many infected individuals are either asymptomatic or have mild flulike symptoms, and these cases might not be diagnosed or reported to the authorities.

Though easily manageable if recognized in time, the disease can still be lethal, which is why accurate diagnostics and proper treatment are essential. The recommended treatment is intravenous penicillin or ceftriaxone [23]. Our patient responded well to both piperacillin-tazobactam and the subsequent week of ceftriaxone intravenously.

This risk of acquiring leptospirosis from pet rodents should be followed closely, especially because pet rodents have become more popular over the past few decades [24]. Being a pet rat owner is highly associated with risk of acquiring leptospirosis [25]. We never found the source of infection, and how our patient’s mouse was infected therefore remains unknown. Pet rodents bought in pet shops should be disease-free, which is why there may be a future need for screening pets for zoonosis, such as leptospirosis. One minor possible intervention could be to provide informational material about how to reduce transmission likelihood to new pet rodent owners, as also suggested by others [14].

## Conclusions

This case report underlines that pet rodents may be sources of leptospirosis. Our findings emphasize that leptospirosis meningitis should be considered in patients with meningeal symptoms who have pet rodents. Obtaining a thorough medical history is essential, including information on animal exposure as well as establishing a timeline of the patient’s symptoms in order to use the proper diagnostics at the right time.

## Data Availability

Not applicable.

## Abbreviations

CRP: C-reactive protein
CSF: Cerebrospinal fluid
MAT: Microagglutination test
PCR: Polymerase chain reaction
WBC: White blood cell count
